# How long do the Hong Kong Chinese expect their URTI to last? – Effects on antibiotic use

**DOI:** 10.1186/s12890-015-0018-y

**Published:** 2015-03-15

**Authors:** Tai Pong Lam, Kwok Fai Lam, Yuk Tsan Wun, Kai Sing Sun

**Affiliations:** Department of Family Medicine and Primary Care, The University of Hong Kong, 3/F, Ap Lei Chau Clinic, 161 Main Street, Ap Lei Chau, Hong Kong; Department of Statistics and Actuarial Science, The University of Hong Kong, Pokfulam, Hong Kong

**Keywords:** Antibiotic use, Chinese, General practice, Expected URTI duration

## Abstract

**Background:**

Recent literature shows that there is a large mismatch between the US patients’ expected duration of acute cough illness and the actual duration. It has been suggested that this discrepancy may lead to antibiotic misuse. Currently, there is limited relevant information for the Chinese. This study aims to investigate the duration that Hong Kong Chinese expect their upper respiratory tract infection (URTI) to last and its possible association with antibiotic use.

**Methods:**

A cross-sectional telephone questionnaire survey with 2,471 adult respondents was conducted in Hong Kong between November and December of 2010. The expected URTI duration of the respondents and their antibiotic use behaviors were analyzed. Multivariable logistic regression analysis was used to adjust for the effects of demographic factors including age, gender, education and income.

**Results:**

Excluding 80 uncertain responses, 544 (23.1%) respondents expected their URTI to last for 1–3 days in general, 613 (25.5%) for 4–6 days, 1168 (48.6%) for 1–2 weeks, and 66 (2.7%) for > 2 weeks. The mean of expected duration was 7.4 (SD:4.2) days. Respondents expecting 1–3 days duration were least likely to ask for and be treated with antibiotics. The proportion of respondents being treated with antibiotics for the last URTI increased from 10% for the 1–3 days group to 23% for the > 2 weeks group (χ^2^ = 19.086, P < 0.001). The effect of expected duration remained significant (P = 0.0188) after adjusting for the effects of demographic factors.

**Conclusions:**

The Hong Kong Chinese expect their URTI to last for about 7 days on average. Different from the notion that underestimation of the actual duration would lead to antibiotic misuse, this study shows that patients expecting a longer duration have a doubled chance to be treated with antibiotics.

## Background

Upper respiratory tract infection (URTI) is the most common illness that sends patients to their doctors [1,2]. Many of them are treated inappropriately with antibiotics. This is partly due to the widely held misconceptions by some doctors about the usefulness of antibiotics for some common respiratory tract symptoms [3,4]. It is also strongly related to patients’ expectations for antibiotic therapy and the doctors’ culture of prescribing [5]. Many studies have shown that some patients expect antibiotic prescriptions for their URTIs and some doctors would prescribe antibiotics based on non-biomedical reasons to satisfy the patients [5-7].

Knowledge and attitudes about URTIs are known to affect patients’ behaviors on antibiotic use [8]. The public generally have little knowledge of the pathology of URTIs. A large proportion of them are confused with the terms “bacteria” and “viruses” [9] and believe antibiotics work well against infections caused by these two agents [10,11]. Besides, the misconception that antibiotics are effective for sore throat, acute cough and purulent nasal discharge are often found to be associated with their desire for antibiotics [10,12,13]. They may also have an inaccurate understanding of the natural history of URTIs. A recent study showed that there was a large mismatch between the US patients’ expectations regarding the average duration of acute cough illness (7–9 days) and the actual mean duration based on a systematic review of the literature (17.8 days) [14]. It has been suggested that efforts to reduce inappropriate antibiotic use should pay attention to this discrepancy which may lead some patients to request antibiotics when the symptom lasts longer than expected [14,15].

Currently, there is a lack of information in the literature about the expected duration of a general URTI episode among the Chinese, who hold different health attitudes and beliefs from the Westerners [16-19]. Our study aimed to explore the expected URTI duration of the Hong Kong Chinese and its possible association with antibiotic use. As the Chinese, either living within or outside China, represent one fifth of the world’s population, many doctors around the world have the chance to look after Chinese patients. The findings of this study would have significant clinical implications to their practice.

## Methods

### Study design and participants

This is part of a larger study on the general public’s knowledge, attitudes and practice with antibiotics. The findings of other study themes have been reported elsewhere [11,13]. A questionnaire for a territory-wide telephone survey in Hong Kong was designed based on the literature and our published findings from in-depth focus group discussions conducted at an earlier stage [11,13]. Prior to pilot-testing with 50 randomly selected household telephone users and subsequent revision, the face- and content-validity of the questionnaire was also tested by academic tutors in family medicine. The responses to the items in the questionnaire mainly had the form of yes (agree), no (disagree), uncertain or refusal. The particular question that helped to determine the respondents’ expected URTI duration was: “How long do you expect one would take to recover from URTI in general?” The given choices were 1–3 days, 4–6 days, 1–2 weeks, and > 2 weeks, with additional options of uncertain or refusal.

The Social Sciences Research Centre of the University of Hong Kong, with the expertise in telephone survey, made random calls to households in the evenings in November and December of 2010. All the 30 interviewers were trained and completed standard practice interviews before the survey to minimize interviewers’ effect in affecting responses. Computer software randomly selected numbers from the latest residential telephone directory. The inclusion criteria were residents speaking local dialect, Cantonese, and aged 18 years or above. In a successful call, the household member aged 18 years or above with the next birthday was invited to take part in the survey. Persons with communication difficulties were excluded. A maximum of five attempts were made for unanswered lines. Ethics approval of this study was obtained from the Institutional Review Board of The University of Hong Kong/Hospital Authority Hong Kong West Cluster (Ref No. UW 07–359).

### Statistical analysis

The outcome measures of the questionnaire survey would be mainly in term of the proportion of a certain characteristics of the general public. In the calculation of the sample size, we would like to control the estimation error of a proportion p to be at most 0.02 (that was the difference between the estimated p and the true value of p) with a probability of at least 0.95. The most conservative value of the sample size n would be obtained by assuming that the true value of p to be 0.5 that required a sample size of n = 2401 [20]. Bivariate analysis by Pearson Chi-squared test was used to determine whether the nominal responses were associated with the choices of expected URTI duration. Multivariable logistic regression analysis was used to estimate the effect of the expected URTI duration (in its ordinal form) by appropriately adjusting for the effects of demographic factors including age, gender, education and income. Wald’s test was used to determine the significance of the factor as a whole. A P-value <0.05 was taken as statistically significant.

We grouped the respondents into three age-groups (<40, 40–65, >65 years) and three household income-groups i.e. low (<HK$10000 (US$1282) (per month), middle (HK$10000-24999 (US$1282 - 1923)), high (≥HK$25000 (US$3205)). The mean of expected URTI duration was estimated by taking one half of the given ranges (2 days for range 1–3 days, 5 days for 4–6 days, 10.5 days (1.5 weeks) for 1–2 weeks, and 21 days for > 2 weeks, taking 4 weeks as maximum [21]). The uncertain and the refusal answers were treated as missing values.

## Results

### Participants recruited

Of the 3,996 successful calls made to households, 376 contacts did not meet the inclusion criteria. Of the remaining 3,620 calls, 813 refused and 336 did not complete the interview, leaving 2,471 completed interviews (response rate 68.3%) for analysis. The age distribution of the respondents was comparable to the Hong Kong population in the 2010 Census.

### Overall responses on the expected URTI duration

Out of the 2,471 respondents, 80 were uncertain on the question regarding their expected URTI duration. Excluding these uncertain responses, 544 (23.1%) respondents expected their URTI to be recovered in 1–3 days, 613 (25.5%) in 4–6 days, 1168 (48.6%) in 1–2 weeks, and 66 (2.7%) over 2 weeks. The median duration was 1–2 weeks, and the mean duration estimated from taking the mid-point of the given ranges was 7.4 (SD:4.2) days.

### Relationship between the demographics and the expected URTI duration

We analyzed the relationship between the respondents’ demographics and their expected URTI duration (Table 1). Bivariate analysis showed that gender (χ^2^ = 30.668, P < 0.001), education (χ^2^ = 32.506, P < 0.001) and income (χ^2^ = 16.577, P = 0.011) had significant effects on their choices. Males and respondents with lower education level were more likely to expect a shorter URTI duration. A larger proportion of high income group expected the duration to be 4–6 days.

**Table 1:** **Relationship between the demographic background and the expected URTI duration**

	**1-3 days n (%)**	**4-6 days n (%)**	**1-2 weeks n (%)**	**> 2 weeks n (%)**	**Total n (%)**	**χ** ^**2**^ **test**
**Age**						
<40 year	142 (20)	187 (26)	364 (51)	18 (3)	711 (100)	χ^2^ = 8.722
40-64 year	320 (24)	346 (26)	634 (48)	36 (3)	1336 (100)	P = 0.190
≥65 year	73 (24)	65 (22)	149 (50)	12 (4)	299 (100)	
**Gender**						
Male	239 (29)	214 (26)	354 (43)	25 (3)	832 (100)	χ^2^ = 30.668
Female	305 (20)	399 (26)	814 (52)	41 (3)	1559 (100)	P < 0.001
**Education**						
Primary or below	123 (29)	82 (20)	201 (48)	15 (4)	421 (100)	χ^2^ = 32.506
Secondary	292 (24)	330 (27)	561 (46)	33 (3)	1216 (100)	P < 0.001
Tertiary	125 (17)	192 (27)	389 (54)	17 (2)	723 (100)	
**Income**						
<HK$10,000	94 (25)	89 (23)	183 (48)	18 (5)	384 (100)	χ^2^ = 16.577
$10,000-24,999	160 (24)	163 (24)	340 (50)	18 (3)	681 (100)	P = 0.011
≥$25,000	159 (21)	224 (30)	359 (48)	13 (2)	755 (100)	

Some data in the categories were missing due to respondents’ refusal to answer. Uncertain responses are excluded.

### Relationship between the expected URTI duration and antibiotic use

Significant differences between the four duration groups (1–3 days, 4–6 days, 1–2 weeks, and > 2 weeks) were observed regarding their antibiotic use behaviors which included asking for antibiotics (χ^2^ = 13.833, P = 0.003), being treated with antibiotics (χ^2^ = 19.086, P < 0.001) and finishing the full course of antibiotics (χ^2^ = 9.968, P = 0.019). As shown in Table 2, around 8-9% of respondents had ever asked for antibiotics in the 1–3 days group, 4–6 days group and 1–2 weeks group, but it doubled to 22% in the >2 weeks group. A steady increase was shown in the proportion of respondents treated with antibiotics for the last URTI, from 10% in the 1–3 days group to 23% in the > 2 weeks group. Speaking of finishing the full course of antibiotics (for URTI and other illnesses), only 64% of respondents in the 1–3 days groups had always complied with it while the other 3 groups were all around 70-72%. After adjusting for the effect of demographic factors (age, gender, education and income) by multivariable logistic regression, the effect of expected URTI duration remained significant on their behaviors in asking for antibiotics (P = 0.0460) and being treated with antibiotics (P = 0.0188). However, the effect was insignificant regarding finishing the full course of antibiotics (P = 0.1226). The estimates for the regression parameters indicated that respondents with shorter expected recovery duration were more likely to give a “NO” response to these questions irrespective of the significance of the factor.

**Table 2:** **Relationship between the expected URTI duration and antibiotic use**

	**Bivariate analysis**					**Multivariable logistic regression P-value**				
	**1-3 days Yes, n (%)**	**4-6 days Yes, n (%)**	**1-2 weeks Yes, n (%)**	**>2 weeks Yes, n (%)**	**χ** ^**2**^ **test**	**Duration** ^**#**^	**Age**	**Gender**	**Education**	**Income**
Have you ever asked the doctor to prescribe antibiotics for yourself?	42 (8)	52 (9)	104 (9)	14 (22)	χ^2^ = 13.833	0.0460	0.5924	0.5276	0.2803	0.5486
					P = 0.003					
Did not ask but expected antibiotics*	104 (22)	103 (19)	245 (24)	10 (22)	χ^2^ = 5.426	0.5614	0.7828	0.6040	0.1850	0.0550
					P = 0.143					
The last time you had a URTI, were you treated with antibiotics?	50 (10)	72 (13)	182 (17)	14 (23)	χ^2^ = 19.086	0.0188	0.7211	0.4590	0.3790	0.0487
					P < 0.001					
Have you ever bought antibiotics over the counter?	43 (8)	48 (8)	88 (8)	9 (14)	χ^2^ = 3.164	0.3951	0.0727	0.8229	0.1564	0.6189
					P = 0.367					
When you were prescribed antibiotics in the past, how often did you finish the full course on time? (always = Yes, other choices = No)	294 (64)	384 (70)	782 (72)	39 (71)	χ^2^ = 9.968	0.1226	<0.0001	0.0026	0.0300	0.1938
					P = 0.019					

Uncertain responses are excluded.

#Duration = expected URTI duration.

*Respondents who had asked for antibiotics did not answer this question.

## Discussion

To our knowledge, this may be the first study to investigate the expected URTI duration of the Chinese. The expected URTI duration of our respondents showed a mean of 7.4 days, which is close to the actual duration of 7–10 days reported in Western literatures [21], as well as that of 10 days found in a recent study on Chinese [22]. Compared with the US study on cough [14], our result suggests that the mismatch of expected and actual duration cannot be generalized from cough to all URTI conditions e.g. common cold and influenza, which cause a variety of symptoms. Nonetheless, when considering the 4 duration groups, we noted that 23% of the respondents expected an URTI duration as short as 1–3 days. This reflects an underestimation of the actual duration by many respondents, who were more likely to be males or with lower education level.

In contrast to the notion that the underestimation of the actual duration would lead to inappropriate antibiotic use [14,15], we found that the 1–3 days duration group used least antibiotics for URTI. In fact, the percentage of respondents asking for or being treated with antibiotics increased significantly with their expected URTI duration. Being treated with antibiotics for the last URTI increased steadily from 10% in the 1–3 days group to 23% in the > 2 weeks group, and the effect of expected duration remained significant after adjusting for the effects of demographic factors. Their expected URTI duration could be based on actual experiences or hypothetical situations. Both experience and imagination of a longer URTI duration might be associated with a perception of having a more severe illness. This could be an explanation for the greater demand for antibiotics. Our finding provides new implication for reducing antibiotic use which has not been addressed by previous mass campaigns [23-26]. It suggested that patients anticipating a longer duration of an infection might expect to have antibiotics regardless of whether the medication was indicated. Thus, the patients’ concept on the duration of an infection is an important determinant of their behavior regarding antibiotics and should be dealt with in patient education.

Similar to the overall rate of antibiotic prescription for URTIs of 23.7% reported in a previous local study [27], the rate of 17.0% found in the current study was also relatively low compared to those (about 35 - 50%) reported by most previous studies in the US, UK and Australia using the national data up to 2006 [28-30]. Probably, as expressed by the participants of our focus-groups at an earlier stage of this study [11], doctors in Hong Kong were getting increasingly stringent with the use of antibiotics. The antibiotic use rate would be higher in Mainland China where antibiotics can be obtained much more easily [31].

### Limitations

This study has some limitations. Firstly, the study findings were based on self-reported data from the respondents. Nevertheless, potential recall bias should be minimal as the questions were asking about their attitudes, usual practice or the most recent experiences. Secondly, the findings of this study are based on the responses of Hong Kong Chinese whose health attitudes and beliefs might be different from people of other nationalities and Chinese living in other parts of the world. Thirdly, this study focused on the impact of expected URTI duration. Other factors such as health status of the respondents, as well as training and confidence of the doctors, might also play a role in antibiotic prescription.

## Conclusions

This study shows that the median and mean of the expected URTI duration of the Hong Kong Chinese are 1–2 weeks and 7.4 days respectively, and 23.1% expected a duration of 1–3 days only. Different from the notion that the underestimation of the actual duration would lead to inappropriate antibiotic use, our study shows that those expecting a longer duration of their URTIs have a doubled chance to be treated with antibiotics. This provides another new dimension for reducing antibiotic misuse.
